# Discovery of Causative Genetic Variants in Patients with Congenital and/or Developmental Anomalies by Exome Sequencing

**DOI:** 10.3390/genes17080863

**Published:** 2026-07-24

**Authors:** Athina Theodosiou, Ludmila Kousoulidou, Ioannis Papaevripidou, Constantia Aristidou, Angelos Alexandrou, Andrea Hadjipanteli, Christina Votsi, Marios Tomazou, Styliana Menelaou, Demetris Efstathiou, Yiannis Ioannou, Emilia Athanasiou, Elena Papamichael, Violetta Christophidou-Anastasiadou, Sofia Ourani, Paola Evangelidou, George A. Tanteles, Carolina Sismani

**Affiliations:** 1 Department of Cytogenetics and Genomics, The Cyprus Institute of Neurology and Genetics, 2371 Nicosia, Cyprus; 2 Department of Clinical Genetics and Genomics, The Cyprus Institute of Neurology and Genetics, 2371 Nicosia, Cyprus; 3 Department of Bioinformatics, The Cyprus Institute of Neurology and Genetics, 2371 Nicosia, Cyprus; 4 Limassol General Hospital, 4131 Limassol, Cyprus; 5 Pediatric Neurology Clinic, Department of Paediatrics, Archbishop Makarios III Hospital, 2012 Nicosia, Cyprus; 6 Unit for Genetics and Endogenous Metabolic Disorders, Department of Paediatrics, Archbishop Makarios III Hospital, 2012 Nicosia, Cyprus; 7 Department of Paediatrics, Archbishop Makarios III Hospital, 2012 Nicosia, Cyprus; 8 Genetics Clinic, Karaiskakio Foundation, 2032 Nicosia, Cyprus; 9 Department of Basic and Clinical Sciences, University of Nicosia Medical School, 2408 Nicosia, Cyprus

**Keywords:** whole-exome sequencing, clinical exome sequencing, Cyprus, congenital anomalies, neurodevelopmental disorders

## Abstract

**Background/Objectives**: Congenital anomalies and neurodevelopmental disorders frequently co-occur and exhibit substantial genetic and phenotypic heterogeneity, posing a persistent diagnostic challenge. Exome sequencing has become an important first- or second-tier diagnostic tool for these conditions, yet diagnostic yields vary considerably depending on phenotype, ancestry, sequencing strategy, and interpretation, with over half of referrals remaining without a definitive genetic diagnosis. **Methods**: We analyzed data from 692 patients referred to the Department of Cytogenetics and Genomics at the Cyprus Institute of Neurology and Genetics between January 2021 and December 2025 for clinical exome sequencing or whole-exome sequencing as part of the diagnostic work-up for congenital disorders and/or syndromic or non-syndromic neurodevelopmental disorders. **Results**: A total of 134 distinct variants were identified, corresponding to an overall diagnostic yield of 17.9% out of which 52 (38.8%) were novel, and 50 variants (37.3%) were de novo, as expected from the high proportion of severe neurodevelopmental presentations. Missense variants were the most prevalent within our cohort, while chromatin and transcriptional regulator genes constituted the largest functional gene category, followed by variants in collagen-encoding genes. **Conclusions**: This study provides the first systematic, mutational-level characterization of a Cypriot Mendelian disease cohort, establishing a local baseline diagnostic yield and revealing a high proportion of novel variants that reflect the underrepresentation of Eastern Mediterranean populations in global databases. These findings underscore the value of submitting population-specific variants to public repositories and of phenotype-driven reanalysis targeting recurrent gene families, supporting more efficient diagnostics and future precision medicine initiatives in Cyprus.

## 1. Introduction

Congenital disorders, commonly referred to as congenital anomalies (CAs) or birth defects, include structural and functional anomalies arising during prenatal life [1,2]. Structural anomalies involve physical malformations of body parts and organs, such as congenital heart disease, limb or neural tube defects, whereas functional anomalies disrupt systemic biological processes, including the neurological, immune, and endocrine systems [3,4]. Globally, an estimated 6% of babies are born with a congenital disorder, representing a major cause of infant morbidity and mortality worldwide [1,2,3]. Although some birth defects have a genetic basis, others are a product of harmful environmental factors known as teratogens, and others are multifactorial, resulting from a complex interaction of genetic and environmental influences. To this day, the etiology remains unknown for approximately half of all patients with birth defects [3].

Beyond their immediate physical impact, CAs frequently serve as early clinical markers for broader systemic disruptions. They often co-occur with neurodevelopmental disorders (NDDs), a clinically and genetically diverse group of early-onset central nervous system disorders. NDDs primarily include intellectual disability (ID), developmental delay (DD), autism spectrum disorders (ASD), and epilepsy-related neurodevelopmental phenotypes, with a combined estimated prevalence of 3–4% of the global population [5]. In clinical practice, presentations are classified as non-syndromic when they occur in isolation, and syndromic when accompanied by dysmorphic features, structural malformations, or multisystem involvement. Neurodevelopmental phenotypes and structural CAs frequently co-occur within the same patient, compounding the diagnostic complexity [6].

The phenotypic complexity of NDDs reflects a highly heterogeneous genetic background. Large-scale parent–child trio studies have shown that de novo coding variants are major contributors to severe developmental disorders [7], while broader NDD analyses also implicate ultra-rare coding variants, copy number variants, as well as autosomal recessive and X-linked mechanisms [8]. Hundreds of genes have now been implicated in NDD etiology [8,9], converging on core biological processes including chromatin regulation, transcriptional control, synaptic function, RNA splicing, ion channel activity, and neuronal communication [10,11]. The relative contribution of each mutational mechanism varies according to ancestry, consanguinity, family structure, and mode of clinical ascertainment [12] and is particularly important in underrepresented populations, where limited population-specific genomic reference data may affect allele frequency assessment and variant interpretation [13,14].

The genetic component of isolated CAs may differ from that of severe neurodevelopmental or syndromic multisystem disorders. While syndromic presentations are more likely to reflect highly penetrant monogenic or chromosomal etiologies, isolated structural anomalies are often more complex, involving rare variants with incomplete penetrance, copy number variants (CNVs), oligogenic mechanisms, mosaicism, or gene–environment interactions [15,16]. This may explain why diagnostic yields are often lower in isolated congenital phenotypes than in multisystem presentations [17,18].

Exome sequencing (ES), a method for small sequence variant detection in coding regions, has become an important diagnostic approach for individuals with suspected monogenic congenital and neurodevelopmental disorders. It can either target all exons of the genome (whole-exome sequencing—WES) or only focus on clinically relevant genes (clinical exome sequencing—CES). A meta-analysis and multidisciplinary consensus statement demonstrated that ES has a higher diagnostic yield than chromosomal microarray analysis (CMA) for unexplained NDDs, reporting an overall diagnostic yield of 36%, rising to 53% when neurodevelopmental disorders were associated with additional clinical features [19]. In line with this evidence, the American College of Medical Genetics and Genomics recommends that ES or genome sequencing (which includes both coding and non-coding regions) should be considered as a first- or second-tier diagnostic test for pediatric patients with CAs, developmental delay, or intellectual disability [20]. However, diagnostic yield and variant spectrum vary substantially depending on phenotype, cohort selection, ancestry, sequencing strategy, and interpretation pipeline. For example, a large cohort study using CES reported higher diagnostic yields in children with ID (34%) or global DD (32%), whereas isolated ASD was associated with a lower diagnostic yield of 16% [21].

The clinical utility of ES extends beyond NDDs: diagnostic yields of 12.7% for congenital heart defects, 13.8% for brain-related malformations, and 29% for non-immune fetal hydrops have been reported [22], while approximately 14% of families with kidney and urinary tract anomalies harbor identifiable single-gene mutations [23]. Nevertheless, despite these advancements, more than 50% of patients referred for ES still remain without a definitive molecular diagnosis [24], reflecting both the limitations of current analytical pipelines and the gaps in gene–disease knowledge.

Middle Eastern and Eastern Mediterranean populations are currently underrepresented in genomic studies and reference databases [13,14], which may affect allele frequency assessment, variant classification, and the interpretation of rare disease-associated variants in these populations [13]. This issue is particularly relevant in Cyprus, where the genetic profile of the population reflects its Eastern Mediterranean location, historical migrations, and community-specific structure [25,26]. In addition, the founder effect, geographic isolation, and population-specific variants have been documented in Cypriot inherited disorders, further supporting the need for population-aware variant interpretation [27].

Recent work from the Cyprus Genome Project [28] has shown that the Cypriot population harbors substantial local genetic variation, including variants absent from major international databases and others that are rare globally but relatively enriched locally. Such differences are clinically important because they can affect rarity filtering, variant classification, and the recognition of locally relevant disease alleles, thereby influencing diagnostic performance for Cypriot patients [28].

This study reports the molecular findings and clinical utility of a large cohort of 692 patients mainly of Cypriot origin, presenting with neurodevelopmental disorders and congenital anomalies. By characterizing the variant spectrum and genotype–phenotype correlations in this cohort, we aim to determine the molecular diagnostic yield, characterize the spectrum of pathogenic and likely pathogenic variants, evaluate the contribution of variants of uncertain significance where relevant, and describe genes and inheritance patterns in this clinically heterogeneous cohort from an underrepresented Mediterranean region.

## 2. Materials and Methods

### 2.1. Participants and Clinical Information

This study is a cohort analysis of patients referred to the Department of Cytogenetics and Genomics at the Cyprus Institute of Neurology and Genetics (CING) between January 2021 and December 2025 for CES or WES. The patients were referred for diagnostic genomic investigation of congenital disorders and/or syndromic or non-syndromic neurodevelopmental disorders. In total, 692 patients were referred, of whom 70% (482) were analyzed as family trios, 22% (156) as singletons, 1% (7) as parent–child duos, and 7% (47) as quartets (siblings–parents). Clinical data were collected from referral forms provided by the clinicians. To support genotype–phenotype correlations and phenotype-driven variant prioritization, detailed phenotypic characteristics, both as narrative clinical descriptions and standardized Human Phenotype Ontology (HPO) terms, were provided for each patient by the referring clinician. Phenotypes, demographic information, family history, and consanguinity were recorded in our internal database. Prior to ES, most patients had undergone diagnostic investigations including chromosomal analysis, arrayCGH and/or targeted molecular testing where appropriate. CES or WES were performed after these investigations failed to identify a genomic alteration that would fully explain the patients’ phenotype. Written informed consent for diagnostic testing was obtained from all participating patients, parents, or their legal guardians for genetic testing and publication of anonymized results.

#### Clinical Presentation

Patients were presented and grouped into five categories: NDD with congenital anomalies/dysmorphism, isolated NDDs, isolated (single-system) CAs without NDD, multisystem, and epilepsy/NDD. The NDD with congenital anomalies/dysmorphism group included patients with neurodevelopmental anomalies accompanied by other features such as dysmorphisms, cardiac, skeletal, neurological, or metabolic anomalies; the isolated NDD group included patients with neurodevelopmental anomalies only, such as ASD, ID, speech and behavioral abnormalities; the isolated (single-system) congenital anomaly group included patients with a single congenital anomaly or multiple anomalies affecting a single organ/system, without profound neurodevelopmental features; the multisystem group included patients with multiple congenital anomalies affecting multiple organs and systems, without profound neurodevelopmental features; the epilepsy/NDD group included patients with neurodevelopmental anomalies accompanied by epilepsy as the main additional feature. A qualitative observation across the whole cohort was that neurodevelopmental features dominated the referral spectrum. Delayed speech and language development, global developmental delay, autistic behavior, microcephaly, and intellectual disability were the most frequently recorded phenotypes, while motor delay, attention deficit hyperactivity disorder, short stature, and long philtrum were among the most noted secondary features.

### 2.2. Sample Preparation and ES

Genomic DNA (gDNA) was extracted from peripheral blood using the QIAmp DNA Blood Midi Kit (QIAGEN, Hilden, Germany). CES libraries were prepared using the TruSight One sequencing panel (Illumina Inc., San Diego, CA, USA), covering ~4800 clinically relevant genes, whereas WES libraries were prepared using either the Nextera Rapid Capture Exome kit (Illumina Inc.) or the updated Illumina DNA Prep with Exome 2.0/2.5 Enrichment kit (Illumina Inc.), following the manufacturer’s guidelines. Paired-end sequencing of the pooled libraries was performed on a NextSeq 500/550 system or NextSeq2000 (Illumina Inc.). Demultiplexing and adapter trimming were performed in the Illumina software environment either on the cloud (BaseSpace Sequence Hub) or locally.

### 2.3. Quality Control

After completion of each sequencing run, a Dragen enrichment analysis along with a MultiQC analysis [29] was performed for initial validation of the run. Sample-specific passing criteria included the coverage depth over the targeted region (>70X), the percentage of sites in the target region above %20X (set at 85%), as well as uniformity of coverage (>90%). Wherever applicable, a locally installed procedure with the Peddy software package v0.4.2 [30] and the KING toolset v2.3.1 [31] was applied to confirm biological sex and genetic relatedness between the proband, siblings, and the parents, allowing for the identification of possible sample mix-ups.

### 2.4. Bioinformatics Analysis, Filtering, and Classification

#### 2.4.1. Platforms

Fastq files were loaded either into VarSome Clinical (Saphetor SA) or Franklin (by Gennox at the time of analysis—currently by QIAGEN). In both platforms, fastq files were uploaded, followed by alignment and variant calling (using the hg19 reference genome). Both the platforms and their embedded tools underwent frequent version updates.

The VarSome Clinical pipeline included: Sentieon’s implementation of bwa-mem for the alignment of reads and DNAscope caller for single nucleotide variants (SNVs) and small indels variant calling. For CNV analysis, the ExomeDepth algorithm was applied [32] as provided by the VarSome platform. CNV analysis was only applied after 2024, when CNVs were inspected and reported as “indications requiring confirmation with an additional method”. The platform, at the time of analysis, did not provide correlation of CNVs with phenotypes, which may have limited the accurate identification of relevant CNVs. CNVs were inspected based on their pathogenicity, length, and Quality Score (≥50) as recommended by the authors of the ExomeDepth algorithm [32]. Correlations with the patients’ phenotypes were studied by manual inspection of gene content and the corresponding disease databases.

The Franklin platform pipelines included: Alignment of reads was performed with the bwa algorithm and GATK HaplotypeCaller and FreeBayes for SNV and small indel variant calling. CNV detection was performed by the Rainbow algorithm. The platform initially created a unique model by using a cohort of 30 samples from more than one batch using the same kit. SNV and CNV calls were reviewed mainly, but not solely, in the “Workbench,” which integrates variant evidence from multiple sources, prioritizing variants using AI and ACMG evidence scoring, resulting in high-confidence calls related to the phenotype. Use of Franklin was limited to a time interval between March 2024 and October 2024.

#### 2.4.2. Filtering and Variant Interpretation

In all cases, variants were filtered based on the following: population allele frequency from gnomAD (frequency < 0.05, excluding variants that are found in 5% or more of all the cases listed in the gnomAD database) to include potential recessive variants present in the population; passing the predefined filter of quality/confidence filter of VarSome or Franklin; variants falling within the targeted region, including untraslated regions (UTRs) and splicing regions (±10); more than 0.2 allelic balance (excludes the WES variant calls that are based on 20% or less of the total reads for the specific nucleotide position). Variant interpretation was guided by correlation with the patient’s phenotype to assess relevance to the suspected disorder and by segregation analysis when parental DNA was available to determine inheritance patterns. In cases where specific genes (or in silico gene panels) were suggested by clinicians, these were prioritized for analysis.

#### 2.4.3. Classification of Variants

The pathogenicity of each variant was assessed either based on the recommendations of ACGS guidelines published in 2020, or on the ACGS 2024 guidelines, [33] which are both based on the first published ACMG standards and guidelines for the interpretation of sequence variants [34]. Additional recommendations by the US ClinGen Sequence Variant Interpretation (SVI) Working Group were considered, and classification was assisted by the automatic scoring ACMG classification of VarSome Clinical or Franklin. A further set of guidelines specific for the interpretation of CNVs that were released by the ACMG in collaboration with ClinGen were considered [35].

Variants were inspected based on the above criteria, and only those classified as pathogenic, likely pathogenic, or variants of uncertain significance (VUS) were reported; VUS were only reported if they had strong evidence of pathogenicity and were associated with the phenotype of the patient.

### 2.5. Variant Validation

Low-confidence SNVs and small indels were validated by bidirectional Sanger sequencing with specifically designed primers (Metabion—Munich, Germany) through the Primer3 web interphase tool flanking the variant site [36]. The applied Biosystems™ BigDye™ Terminator v1.1 Cycle Sequencing Kit (ThermoFisher Scientific—Waltham, MA, USA) and Affymetrix ExoSAP-IT^®^ (Fisher Scientific—Pittsburgh, PA, USA) were used for this procedure. Segregation analysis was performed in available relatives and, when appropriate, especially for singletons. In selected unresolved cases, additional reanalysis was undertaken where clinically justified.

For CNV validation, quantitative real-time polymerase chain reaction (qRT-PCR) was performed using SsoFast™ EvaGreen^®^ Supermix (Bio-Rad Laboratories, Inc., Hercules, Contra Costa County, CA, USA) and specifically designed primers (Metabion, Munich, Germany). Amplification and fluorescence detection were carried out using the CFX96 Real-Time PCR Detection System (Bio-Rad Laboratories, Inc., Hercules, Contra Costa County, CA, USA).

## 3. Results

### 3.1. Cohort Composition

**Figure 1:**
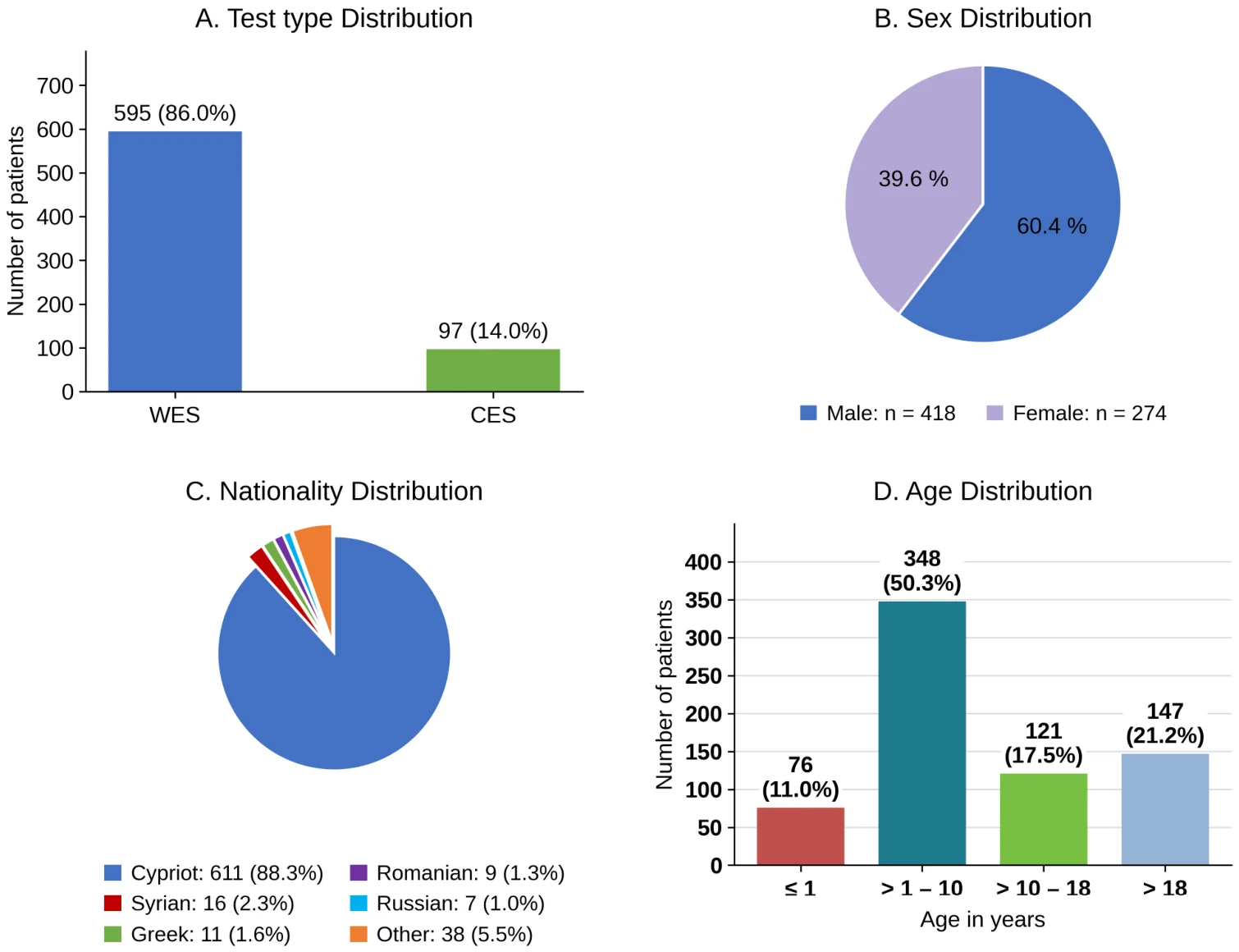
Cohort overview (*n* = 692 patients). (**A**) Test type distribution: WES, Whole Exome Sequencing; CES, Clinical Exome Sequencing. (**B**) Sex distribution. (**C**) Nationality distribution. (**D**) Age at genomic evaluation.

A total of 692 patients underwent ES during the study period. An overview of the cohort structure is presented in Figure 1A–D. Two test types were employed: WES (*n* = 595, 86.0%) and CES (*n* = 97, 14.0%) (Figure 1A). The cohort was predominantly male, comprising 418 males (60.4%) and 274 females (39.6%) (Figure 1B).

The majority of patients were of Cypriot origin (*n* = 611, 88.3%). Syrian nationals constituted the second most frequent group (*n* = 16, 2.3%), followed by Greek (*n* = 11, 1.6%), Romanian (*n* = 9, 1.3%), and Russian (*n*= 7, 1%) patients. The remaining individuals represented more than twenty additional nationalities (combined *n* = 38, 5,5%) (Figure 1C).

The age distribution was broad, encompassing infants, children, and adults, and spanned a range from 29 days to 71.0 years (median 8.0 years, IQR 4.0–15.3). The majority of patients were pediatric at referral (*n* = 545, 78.7%). A substantial number of adults (>18 years) (*n* = 147, 21.3%) were also included, comprising individuals with longstanding undiagnosed conditions or those not referred for testing at an earlier stage. The age distribution of the cohort is summarized in Figure 1D.

### 3.2. Diagnostic Yield and Genomic Findings Overview

Out of 692 patients evaluated, genomic findings were obtained in 117 unique families/probands; these collectively comprised 134 variants distributed across 109 distinct disease-associated genes. The variant total exceeds the case total because 16 cases included more than one causative variant (14 cases with two variants and 2 cases with three variants). These additional variants arise from compound heterozygosity at recessive genes, variants in two functionally independent disease genes in the same patient, and a complex in-cis intronic event.

Because several findings were detected in two or more affected siblings within the same family, the 117 family-level findings corresponded to 124 affected individuals carrying a finding.

Restricting the diagnostic yield calculation to cases having at least one pathogenic (P) or likely pathogenic (LP) variant (a strict definition), a molecular diagnosis was established in 111 of 692 patients (16.04%). A further 1.9% (13 patients) were resolved based solely on one or more variants of uncertain significance (VUS). When these cases are included (a broad definition), the cumulative yield rises to 124 of 692 (17.9%). Diagnostic rates were also estimated separately for each of the broad clinical referral categories and are summarized in Table 1. The highest rate was observed in the epilepsy/NDD group, in which 5 of 10 cases were solved (50.0%), although this category included a very small number of individuals. Among the larger referral groups, multisystem presentations and isolated (single-system) congenital anomalies yielded comparable rates of 22.6% (28/124) and 21.5% (29/135), respectively. Neurodevelopmental disorders (NDD) accompanied by congenital anomalies or dysmorphism—the largest referral group—showed a diagnostic rate of 16.4% (57/347), whereas isolated NDD had the lowest yield, at 6.6% (5/76).

### 3.3. Variant Architecture, Novelty, Classification, and Inheritance

**Figure 2:**
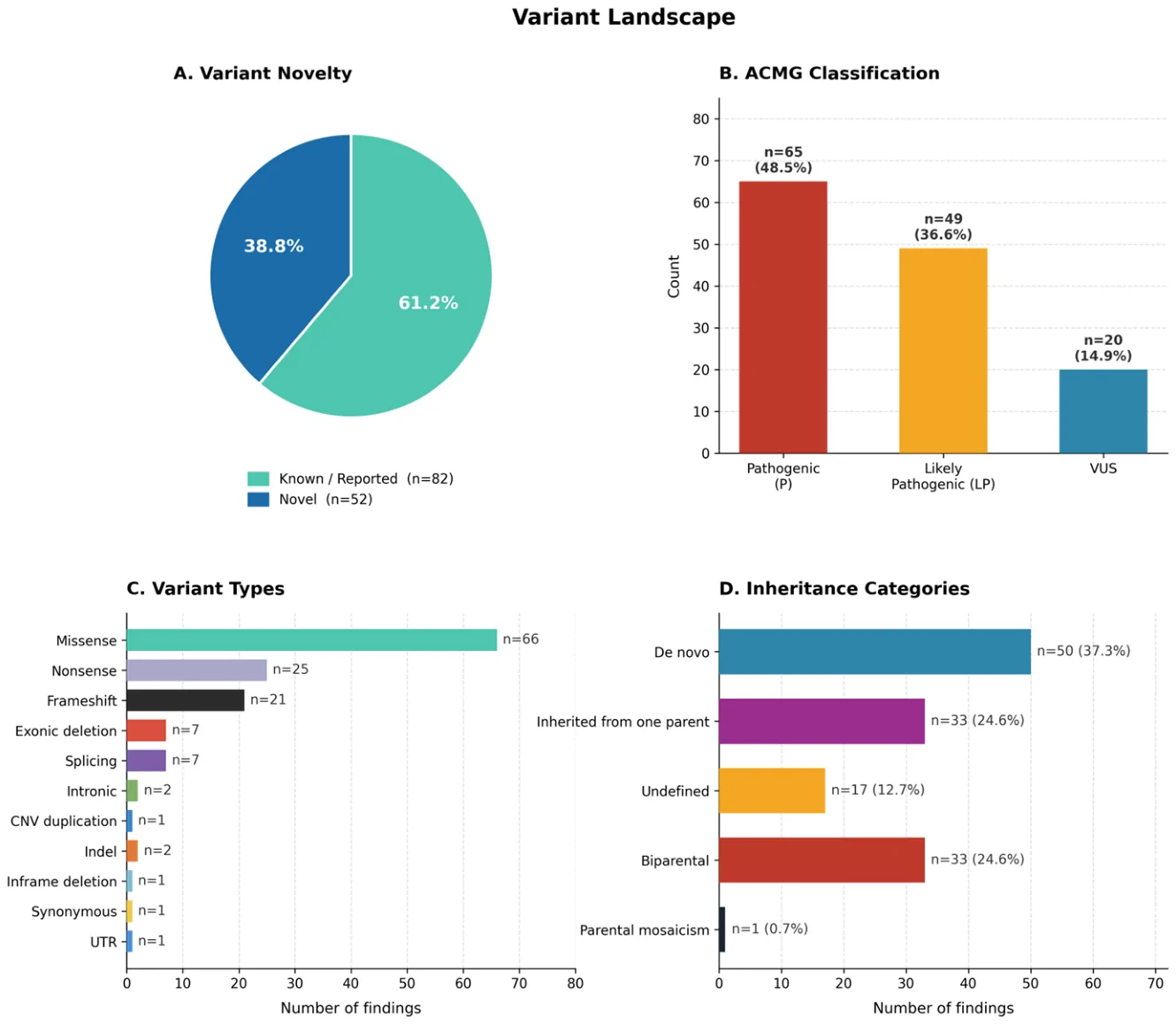
Variant landscape across the 134 reported variants. (**A**) Variant novelty (Novel = no prior entry in ClinVar or dbSNP at the time of reporting; Known/Reported = previously documented). (**B**) ACMG/AMP variant classification (P, Pathogenic; LP, Likely pathogenic; VUS, Variant of uncertain significance). (**C**) Variant types. (**D**) Inheritance categories.

An overview of the variant characteristics is presented in Figure 2. Of the 134 reported variants, 52 (38.8%) had no prior entry in local datasets, ClinVar, or dbSNP at the time of reporting and were therefore designated novel, whereas 82 variants (61.2%) had been previously reported (Figure 2A). Novel variants are presented in Table 2, and the remaining known/previously reported variants are presented in Supplementary Table S1. All variants were submitted to ClinVar with accession numbers: SCV007615186-SCV007615318.

All variants were assessed using the ACMG/AMP evidence-based framework and ACGS guidelines [33], considering the allele frequency data from gnomAD, computational predictors, ClinVar submissions and reports in the literature, available functional studies, and segregation data where parental samples were available. Of the 134 variants for which sufficient evidence allowed a categorical classification, 65 were pathogenic (P; 48.5%), and 49 were likely pathogenic (LP; 36.6%), together accounting for 85% of all classified variants. The remaining twenty variants (14.9%) were classified as variants of uncertain significance (VUS) (Figure 2B). Nine VUS were heterozygous standalone findings in autosomal-dominant disease genes in which parental samples were absent or uninformative. Seven VUS were detected in compound heterozygosity with a confidently classified P or LP allele in autosomal-recessive genes. The remaining four VUS were recessive findings (homozygous or compound heterozygous).

Missense variants dominated the molecular landscape, accounting for 66 of 134 variants (49.2%), consistent with the expected output of protein-coding sequence capture. Loss-of-function classes collectively contributed 53 variants: nonsense (*n* = 25, 18.6%), frameshift (*n* = 21, 15.6%), and splicing (*n* = 7, 5.2%). Structural copy number variants, exonic deletions (*n* = 7, 5.2%), and one duplication (0.7%), comprised 5.9% of all variants. The remaining findings included two indels (1.5%), two intronic variants in cis configuration (1.5%), one in-frame deletion, one synonymous variant with demonstrated splicing consequences, and one 3-UTR regulatory variant. The complete distribution is shown in Figure 2C.

Variant inheritance was determined exclusively through parental testing and segregation analysis, since most cases were investigated as family trios (Figure 2D). De novo origin was shown for 50 of 134 variants (37.3%), as expected from the high proportion of severe neurodevelopmental presentations in the referral base. Variants transmitted from one parent (maternal or paternal) accounted for 24.6% (*n* = 33), including X-linked inheritance. Biparental transmission, encompassing compound heterozygous and homozygous autosomal-recessive variants, was confirmed in 33 variants (24.6%). For 17 variants (12.7%), the second parent was not tested, either because the patient was referred as a singleton or because only one parent was available and therefore the inheritance could not be formally established, and this category is labeled ‘Undefined’ in Figure 2D.

**Figure 3:**
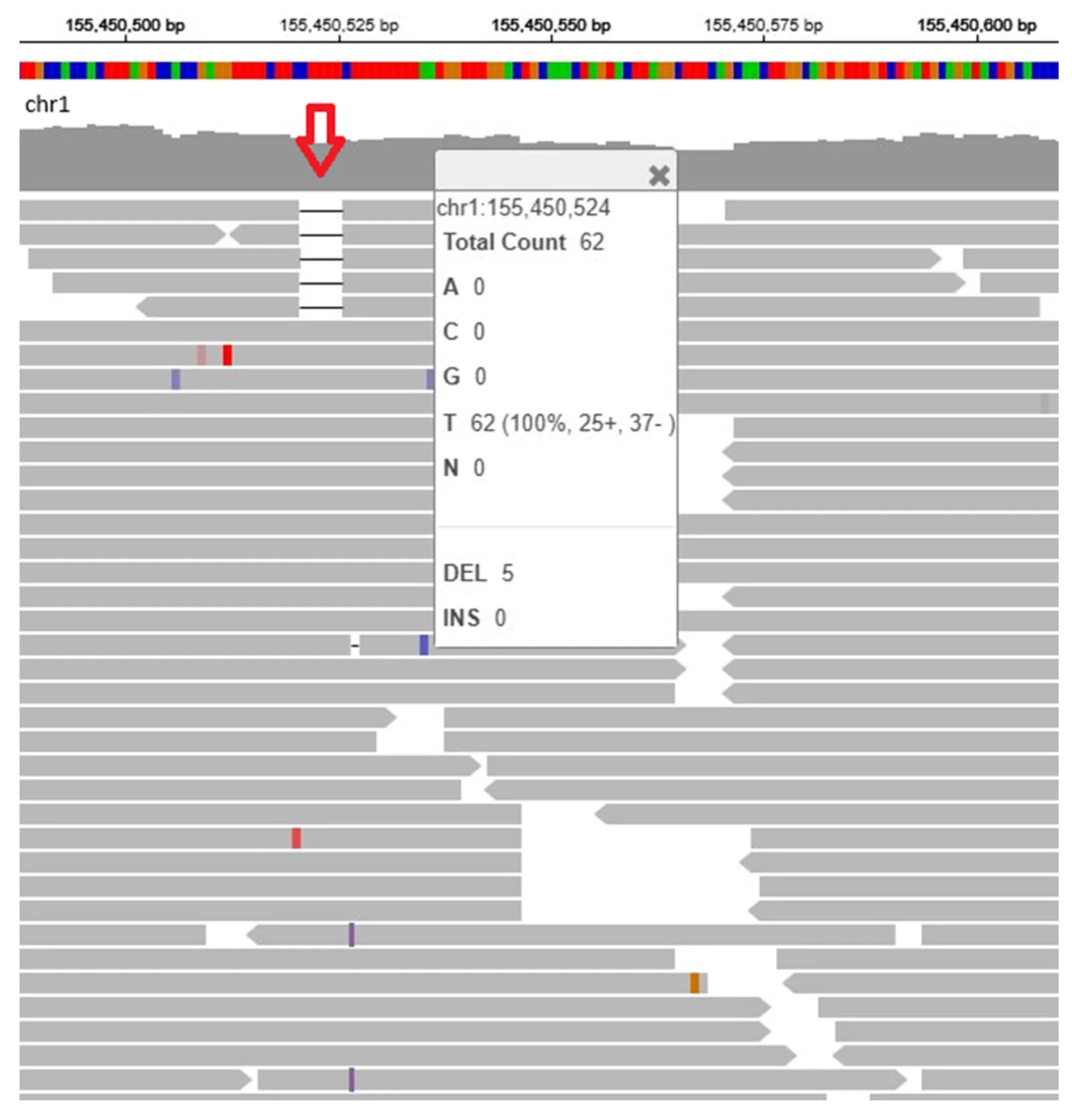
IGV illustration of paternal buccal swab WES results demonstrating gonadal mosaicism of frameshift variant (shown with the red arrow) in *ASH1L* gene (allele fraction 5/62, 8%).

An apparently de novo frameshift variant in the *ASH1L* gene NM_018489.3:c.2136_2140del p.(Gly714LysfsTer6) arose in the context of parental germline mosaicism. An affected sibling of the proband was shown to carry the same variant, which warranted targeted parental buccal swab testing, resulting in detection of paternal mosaicism (Allele fraction: 5/62, ~8%) (Figure 3).

### 3.4. Gene Distribution, Recurrent Findings, and Inheritance Mode

**Figure 4:**
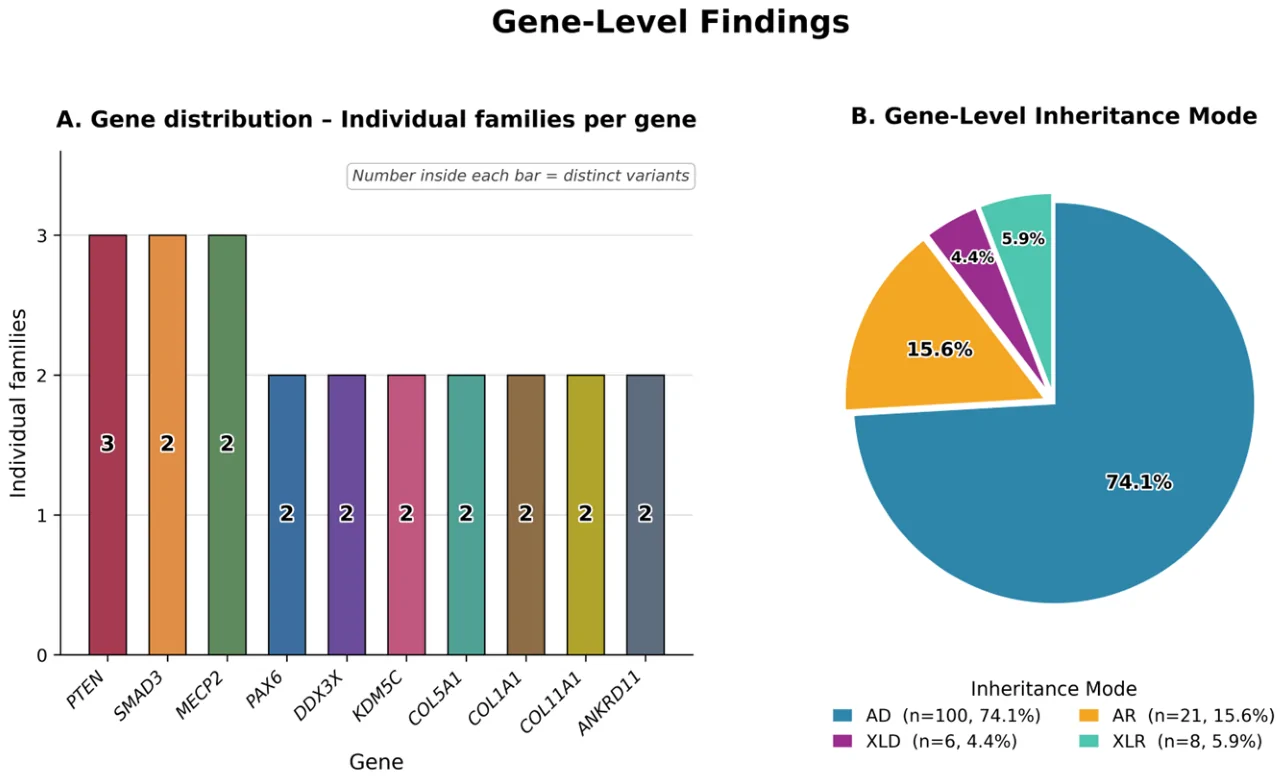
Gene-level findings. (**A**) Recurrent genes (≥2 families), with y-axis labels showing number of individual families. The number of distinct variants observed in each gene is shown inside each bar; the single-patient gene variants are excluded from this figure. (**B**) Gene-level inheritance mode at the patient level: AD, autosomal dominant; AR, autosomal recessive; XLD, X-linked dominant; XLR, X-linked recessive.

The variants detected in 117 cases mapped to 109 unique disease genes. Twenty-one genes harbored variants in two or more individuals when sibling cases were counted (Figure 4A). The highest number of distinct variants was found in the *PTEN* gene (three variants in three unrelated families). *PTEN* findings were found in patients with macrocephaly and autism spectrum disorder with variable severity, consistent with the broad expressivity of *PTEN* hamartoma tumor syndrome. Two *SMAD3* variants were detected in three unrelated families. Similarly, two *MECP2* variants were detected in three unrelated families. Two distinct variants in *PAX6*, *DDX3X*, *KDM5C*, *COL5A1*, *COL1A1*, *COL11A* and *ANKRD11* were found in two unrelated families. The remaining findings were single variants detected in families with one or more affected individuals.

Autosomal dominant (AD) disease genes accounted for the majority of findings (100 individuals, 74.1%), largely reflecting the de novo mutational burden associated with severe neurodevelopmental presentations. Autosomal recessive (AR) genes contributed 15.6% (21 individuals), with compound heterozygosity being the predominant AR mechanism and homozygosity accounting for the remainder. X-linked dominant (XLD) and X-linked recessive (XLR) genes contributed 6 (4.4%) and 8 (5.9%) individuals, respectively (Figure 4B).

### 3.5. Dual-Gene Findings

Four cases (3.4% of resolved cases) involved variants in two functionally independent disease genes within the same patient, suggesting a possible dual diagnosis. These included: a patient carrying a de novo pathogenic stop-gain variant in the *KMT2A* gene together with biparentally inherited likely pathogenic frameshift variants in the *ZNF469* gene (compound heterozygous), resulting in concurrent Wiedemann–Steiner syndrome and Brittle Cornea syndrome-1; a patient with a maternally inherited likely pathogenic missense variant in *PTEN* and a paternally inherited likely pathogenic nonsense variant in *KDM4B*; a patient with novel a likely pathogenic frameshift variant in *FBXO11* and a VUS nonsense variant in *LMBRD2*; and a patient with a pathogenic missense variant in *GJB2* and a missense VUS in the *COL11A1* gene.

## 4. Discussion

### 4.1. Overview

In this study, we report the molecular findings of 692 patients referred for exome sequencing (ES) to the Cytogenetics and Genomics Department at the Cyprus Institute of Neurology and Genetics during 2021–2025. The majority of referred patients were of Cypriot origin, male, and pediatric at referral, with neurodevelopmental features dominating the clinical spectrum. The overall diagnostic yield was 16.04% under a strict definition (including only pathogenic or likely pathogenic variants) and 17.9% when variants of uncertain significance (VUS) with strong supportive evidence were included. While early large-scale trio studies reported higher yields of 25–40% [23,37,38,39] our results align closely with more recent unselected clinical cohorts, particularly those enriched for isolated ASDs or CAs, with diagnostic yields between 15 and 19.6% [40,41].

In general, the detection rate is influenced by cohort structure, ancestry composition, sequencing strategy, variant-filtering thresholds, and the timing of data curation. Our cohort is highly enriched for isolated or less specific neurodevelopmental presentations, which generally have lower diagnostic yields than syndromic or multisystem phenotypes. For example, a previous study reported a 31.5% yield for syndromic GDD/ID, but only 6.1% for ASD and 7.1% for other isolated NDDs [42]. In contrast, an earlier study performed by our group applied strict phenotypic inclusion criteria and focused on patients with multiple malformation syndromes and at least one neurodevelopmental anomaly, after extensive negative prior investigations, achieving a diagnostic yield of 43.2% [43]. This demonstrated that strict inclusion criteria for patients with well-defined, multisystem phenotypes and early-onset severe presentations, rather than isolated NDDs, substantially increase the prior probability of an identifiable monogenic cause and thus the overall diagnostic yield.

Standard exome pipelines, such as the one used in this study, cannot reliably detect complex indels, repeat expansions, and certain structural variants. Our patients were evaluated using ES, whereas complementary testing methods such as whole-genome sequencing, RNA sequencing, methylome/epigenetic profiling, long-read sequencing, and optical genome mapping have been shown to capture variant classes and mechanisms missed by standard exome analysis [19,44].

The diagnostic yield is also influenced by the genetic background of the studied cohort. In underrepresented ancestries such as the Cypriot population, the lack of robust population-specific allele frequency reference data can make it more difficult to distinguish truly rare pathogenic variants from benign local polymorphisms, thereby increasing diagnostic uncertainty [28]. Because Cyprus has a distinctive genetic background shaped by historical migrations, population substructure, and founder effects, reliance only on large international databases may be insufficient for optimal variant interpretation in this setting [26,28]. Local founder variants and population-specific disease patterns warrant ancestry-matched reference data when interpreting rare variants [45,46], as limited Cypriot-specific reference data may contribute to the under-classification of potentially causative variants, lowering the overall reportable yield.

Fifty-two of 134 variants (38.8%) were absent from both local datasets, ClinVar, and dbSNP at the time of clinical reporting and were therefore considered novel. The relatively high yield in novel variants in our study may be explained by the fact that the Cypriot population, as a small island population with regional substructure and historically endogamous in some communities, may harbor rare or locally enriched alleles that are poorly represented in large international reference datasets. This effect is likely compounded by the limited representation of Cypriot and, more broadly, Eastern Mediterranean genomes in resources such as gnomAD, ClinVar, and the 1000 Genomes Project, so that several novel variants are detected because of under-ascertainment rather than recent origin. For this reason, systematic submission of the novel variants identified here, particularly those supported as pathogenic or likely pathogenic, would improve variant interpretation and increase the utility of public databases for future diagnostic work in Cypriot and neighboring Mediterranean populations.

### 4.2. Findings

Below we discuss the most significant findings of our study in non-OMIM genes; frequently observed categories of genes such as collagen-related and chromatin/transcriptional regulators; Sodium channelopathies; X-linked neurodevelopmental genes, and the gonadal mosaicism case.

#### 4.2.1. Non-OMIM Genes

Beyond expanding the mutational spectrum of known disease genes, our ES analysis successfully identified candidate variants in genes not yet associated with an OMIM-disease phenotype. Specifically, we identified candidate variants in *KCNJ3* and *KCND2*, two genes encoding critical subunits of potassium channels. These channels are essential for regulating cellular excitability and electrical activity throughout the body, most notably in the heart and brain. Given their critical physiological roles, these variants demand careful consideration as novel causes of NDDs.

The *KCNJ3* candidate variant

We identified a novel de novo variant in the *KCNJ3* gene [NM_002239.4:c.634A>G; p.(Met212Val)] classified as VUS in a patient presenting with neurodevelopmental disturbances and generalized seizures (absences and myoclonic seizures). While *KCNJ3* currently lacks a formalized OMIM disease association, emerging literature strongly supports its role in human pathology. The *KCNJ3* gene encodes the G protein-activated inward rectifier potassium channel 1 (GIRK1), which, together with other GIRK subunits, is vital for maintaining the resting membrane potential and controlling neuronal and cardiac excitability.

Early genetic evidence supporting the association with epilepsy came from Chioza et al. (2002) [47] who investigated potassium channel genes in idiopathic generalized epilepsy and reported a significant association between a *KCNJ3* polymorphism and idiopathic generalized epilepsy, with the strongest signal observed in patients with absence seizures. Large-scale epilepsy sequencing studies have also supported a role for rare variation in epilepsy risk, including enrichment signals involving *KCNJ3* in genetic generalized epilepsy cohorts [48].

Although the original association study provided suggestive rather than definitive evidence, more recent data have strengthened the relationship between *KCNJ3* and epilepsy. In particular, Li et al. identified two de novo *KCNJ3* variants in patients with early-onset epilepsy and showed through electrophysiological analysis that these variants significantly reduced channel activity, supporting a loss-of-function mechanism [49].

These findings suggest that altered *KCNJ3*/*GIRK1* function may contribute to epilepsy by impairing inhibitory potassium currents. However, because the available evidence includes both candidate gene association data and a limited number of rare variant reports, *KCNJ3* should currently be regarded as an emerging or moderate evidence epilepsy gene, with further evidence needed to fully establish causality. Further evidence is needed for reclassification of the variant to likely pathogenic.

The *KCND2* candidate variant

We identified a previously reported (ClinVar ID:1009567) de novo missense variant in the *KCND2* gene [NM_012281.3:c.1208C>T p.(Pro403Leu)]. The gene encodes the voltage-gated potassium channel alpha subunit Kv4.2, which mediates the A-type transient outward potassium current. Although *KCND2* is not yet linked to a disease in OMIM, converging evidence from several independent cohorts supports its critical role in neurodevelopment and epilepsy. Early reports by Lee et al. (2014) [50] identified a de novo heterozygous missense variant (p.Val404Met) in identical twins with infantile-onset refractory epilepsy and autism. This variant is located in the S6 transmembrane domain, which is a critical region for channel gating. Subsequent detailed biophysical analyses demonstrated that this variant results in complex kinetic alterations, including enhanced closed-state inactivation, slowed recovery from inactivation, and decreased closure following activation [51]. More recently, Zhang et al. (2021) [52] expanded the phenotypic spectrum by reporting six unrelated individuals with early-onset global developmental delay, impaired motor and cognitive development, and, in some cases, developmental epileptic encephalopathy and dysmorphisms. All six individuals carried heterozygous missense variants in *KCND2* (including p.Glu323Lys, p.Pro403Ala, p.Val404Leu, and p.Val404Met) affecting sites crucial for channel gating. The aforementioned recent reports strongly support the conclusion that disruptive missense variants in the S6 gating domains of *KCND2* alter channel kinetics and cause the severe epileptic and developmental features observed in these patients, demonstrating the critical role of A-type potassium channels in normal brain development.

#### 4.2.2. Findings in Collagen-Related Genes

Variants in collagen-encoding genes emerged as one of the most robust and informative diagnostic categories observed in our cohort, accurately resolving a diverse spectrum of connective tissue, basement membrane, and skeletal phenotypes. The identification of 13 variants across 10 loci (*COL1A1*, *COL2A1*, *COL4A1*, *COL4A4*, *COL5A1*, *COL9A2*, *COL10A1*, *COL11A1*, *COL11A2*, and *COL18A1*) in 14 individuals underscores the clinical utility of comprehensive genomic sequencing in capturing the broad phenotypic pleiotropy inherent to collagen biology, which spans the skeleton, the ocular and vitreoretinal apparatus, the vasculature, the kidney, and the skin.

In agreement with other studies, we observed distinct pathogenic variant clustering across functional collagen subgroups. For example, fibrillar collagen defects driven by pathogenic variants in *COL1A1* and *COL5A1* reliably predicted classical bone and connective tissue disorders. The *COL1A1* variants segregated with a likely non-deforming osteogenesis imperfecta phenotype, in which the cardinal anomaly is scoliosis accompanied by connective tissue and ocular features of the milder end of the spectrum, namely generalized joint hypermobility, soft and hyperextensible skin, and pes planus. *COL5A1* variants produced classical Ehlers–Danlos syndrome (cEDS), characterized by skin hyperextensibility with atrophic scarring, easy bruising, soft doughy skin, and generalized joint hypermobility with associated musculoskeletal manifestations (pes planus, scoliosis) and blue sclerae. Notably, the identification of a de novo nonsense *COL5A1* variant in a family lacking prior history is consistent with the established mutational epidemiology of cEDS. Recent literature suggests that while roughly 50% of cEDS cases are inherited, de novo occurrences are frequent, emphasizing the necessity of unbiased genomic screening over strict reliance on familial history [53].

Pathogenic variants in *COL4A1* and *COL4A4* correlated with small-vessel and renal disease phenotypes. *COL4A1* defects underlie a systemic angiopathy whose anomalies are principally vascular and neurovascular. In our patient, the neurological picture (spastic paraparesis, epilepsy, and ischaemic lesions on MRI) overlapped this small-vessel phenotype. A patient with *COL4A4* variants presented with isolated persistent haematuria.

As for the cartilage-associated collagen genes (*COL2A1*, *COL9A2*, and *COL11A1*), their diagnostic interpretation proved significantly more challenging as all three VUS identified in our collagen cohort (23% of total collagen variants) fell within this subgroup. The affected individuals in our cohort presented predominantly ocular and craniofacial findings (lens dislocation, facial asymmetry) and articular findings (joint laxity, marked genua recurvata, and slender fingers). This high VUS burden mirrors findings from broader ES studies on skeletal dysplasias, where extreme locus heterogeneity, combined with a lack of robust functional assays for atypical missense variants, severely limits variant reclassification. These findings underscore the critical need for extensive segregation and functional studies to resolve VUS designations in complex skeletal presentations [54].

A patient in our cohort carried two findings, a confirmed pathogenic variant in *GJB2* and a variant of uncertain significance in *COL11A1*. Pathogenic variants in the *GJB2* gene are the most common cause of hearing loss that occurs in isolation, while pathogenic variants in *COL11A1* are typically linked to Stickler syndrome, where hearing loss comes bundled with other features. When both appear in the same patient, it becomes genuinely difficult to know whether two separate conditions co-exist, or whether one variant is affecting the expression of the other. This kind of diagnostic gray zone is not rare. Exome studies increasingly identify patients whose genetic profile does not fit neatly into a single diagnosis, reflecting the growing recognition that epistatic and modifier effects can obscure discrete genotype–phenotype boundaries in complex hereditary conditions.

#### 4.2.3. Sodium Channelopathies

Three patients in our cohort harbored pathogenic missense variants in voltage-gated sodium channel genes: *SCN1A* (NM_001165963.4:c.1177C>T, p.(Arg393Cys); maternally inherited), novel *SCN2A* (NM_001040143.2:c.832T>C; de novo), and *SCN4A* (NM_000334.4:c.4364T>C, p.(Ile1455Thr); parental status not defined). The *SCN1A* and *SCN2A* variants were identified in patients presenting with developmental and epileptic encephalopathies (DEEs), whereas the *SCN4A* variant was found in a patient with paroxysmal weakness indicative of hyperkalaemic periodic paralysis. These findings build upon prior prenatal research from our group [55], in which missense variants were detected in *SCN2A* and *SCN4A* genes. These data expand the known clinical and mutational spectrum of sodium channelopathies within the Cypriot population.

An interesting observation from these findings is that all variants are missense substitutions, including the findings from our previous study [55], affecting highly conserved amino acid residues across paralogs, which is the predominant mutational mechanism driving disease in this gene family. Another intriguing clinical scenario arises from the presence of the *SCN1A* variant in a non-affected individual (the patient’s mother). While *SCN1A* variants are classically associated with severe Dravet syndrome via a de novo, dominant loss-of-function mechanism, a significant proportion of milder phenotypes such as genetic epilepsy with febrile seizures plus (GEFS+) arise from inherited missense variants with reduced penetrance. The clinical presentation of our proband aligns closely with this milder, inherited GEFS+ spectrum [56].

Overall, three sodium-channel variants across a 692-patient cohort (approximately 0.4%) were detected. The diagnostic yield is consistent with published prevalence estimates from comparable large-scale diagnostic series, reinforcing the importance of these loci in routine genomic screening [57].

#### 4.2.4. Chromatin and Transcriptional Regulators (NDD Spectrum)

Eighteen distinct chromatin modifier and transcriptional regulator genes harbored diagnostic findings in our cohort, accounting for 19 variants in 21 patients. This represents the largest functional gene category observed in our study and a defining biological feature of NDDs. The identified variants disrupted genes involved in various functional aspects of epigenetic machinery. These included writers and erasers of histone marks (*KMT2A*, *KMT2D*, *SETD2*, *SETD5*, *ASH1L*, *KDM4B*, *KDM5C*, *KDM6B*), essential components of the SWI/SNF chromatin remodeling complex (*SMARCA2*, *SMARCA4*, *BICRA*), transcriptional co-activators *(CREBBP*), structural cohesin proteins (*NIPBL*), and broad transcriptional regulators (*CHD7*, *CHD8*, *SIN3A*, *ZBTB18*, *ZMYND11*).

The main disease mechanism in this group was haploinsufficiency: loss-of-function variants (nonsense, frameshift, or splice-site) accounted for 13 of 19 findings (68%), and most were de novo. These variants produced a range of disorders including Wiedemann–Steiner syndrome (*KMT2A*), Kabuki syndrome type 1 (*KMT2D*), Coffin–Siris syndrome (*BICRA, SMARCA4*), Nicolaides–Baraitser syndrome (*SMARCA2*), KBG syndrome (*ANKRD11*), Rubinstein–Taybi/Menke–Hennekam-1 (*CREBBP*), Cornelia de Lange syndrome type 1 (*NIPBL*), CHARGE syndrome (*CHD7*), and Witteveen–Kolk syndrome (*SIN3A*). Together, these conditions illustrate how heterozygous, dosage-sensitive variants in chromatin and transcriptional regulators disrupt neurodevelopment, and they highlight the clinical value of WES for children with complex NDDs [58].

Notably, two genes in the epigenetic cluster (*ANKRD11*, *KDM5C*) were each found in more than one unrelated patient, consistent with their relatively high frequency in NDD cohorts. The value of ES in complex, overlapping phenotypes was illustrated by a dual-diagnosis case in which the patient had Wiedemann–Steiner syndrome due to a *KMT2A* variant as well as Brittle Cornea syndrome caused by biallelic *ZNF469* variants. *ZNF469* encodes a zinc–finger transcription factor that helps regulate extracellular matrix production, so this case combines defects in both chromatin-level regulation and connective tissue homeostasis. It was the most clinically complex dual-gene case in our cohort and shows how distinct Mendelian disorders can overlap enough to obscure each other in standard clinical assessment [59]. These dual-gene findings illustrate the importance of not terminating variant interpretation upon identification of a first plausibly causal variant, a principle that becomes especially relevant when the phenotype is more complex than a single Mendelian condition would predict.

#### 4.2.5. X-Linked Neurodevelopmental Disorders

Thirteen variants across ten X-linked neurodevelopmental disorder genes were identified in our cohort. These findings closely recapitulated canonical, sex-specific inheritance and phenotypic patterns widely documented in the literature. The two identified *MECP2* variants (one variant was reported in two separate individuals) included de novo events in three females presenting with classic Rett syndrome, a progressive neurodevelopmental disorder classically driven by female-specific heterozygous loss-of-function, and one distinct variant in a male presenting with X-linked intellectual disability (XLID). Similarly, *DDX3X* variants were identified in a female with developmental delay (missense) and in a male hemizygote (nonsense variant). Additionally, *KDM5C* contributed to the diagnosis of two cases, including a de novo frameshift in a male, which is a relatively rare but previously described mode of inheritance for this typically maternally inherited XLID gene [60].

As expected for X-linked recessive (XLR) conditions, the carrier mother inheritance model accounted for the majority of findings in affected males, representing five of the eight XLR variants. Notable diagnoses within this group included *RPS6KA3* (associated with Coffin-Lowry syndrome), *CUL4B* (causing Cabezas-type syndromic XLID), and *HNRNPH2* (associated with Bain-type XLID, a disorder that predominantly affects females). Furthermore, we identified a missense VUS in *OTUD5*, a gene recently linked to an emerging multiple congenital anomalies-neurodevelopmental syndrome (OMIM# 300713). This VUS is considered a critical target for future segregation analysis to clarify its potential pathogenicity and contribute to the expanding genotype–phenotype correlations for this locus.

#### 4.2.6. From Apparently De Novo to Gonadal Mosaicism

One of the most interesting findings was the identification of the same novel, likely pathogenic, apparently de novo frameshift variant NM_018489.3:c.2136_2140del p.(Gly714LysfsTer6) in two affected siblings (*ASH1L* gene). Heterozygous pathogenic variants in the *ASH1L* gene can cause autosomal dominant intellectual developmental disorder-52 (MRD52). Further targeted investigation of parental buccal swabs demonstrated that this variant was present in a mosaic state of ~8% in the clinically unaffected father (see Figure 3). Parental mosaicism in *ASH1L* has been reported once, by Di Muro et al. (2025), who inferred gonadal/gonosomal mosaicism from recurrence of an intragenic deletion in three siblings with two non-carrier parents [61]. This mosaicism observation illustrates a critical and frequently underestimated limitation of assigning de novo status based only on routine parental testing. If only one of the siblings had been tested in this case, it would have been miscategorized as de novo. Although de novo variants may represent post-zygotic mutational events, they may also arise from low-level parental mosaicism, particularly gonadal mosaicism, which may not be detectable in peripheral blood. This distinction is clinically relevant because parental mosaicism can substantially increase recurrence risk compared to a post-zygotic de novo event. Therefore, recurrence in siblings should prompt consideration of parental mosaicism and, where feasible, sensitive testing of parental tissues or sperm to allow accurate recurrence risk estimation and genetic counseling.

### 4.3. Limitations and Future Directions

A number of limitations in the present study should be acknowledged, mostly derived from the diagnostic nature of the performed experiments. A key limitation is that the cohort consists of clinically referred patients rather than a population-based sample selection, which introduces biases and gaps that may hinder comprehensive interpretation. Specifically, there is a selection bias toward severe pediatric presentations, limiting extrapolation to milder or adult-onset phenotypes. In addition, in approximately 14% of findings, inheritance information could not be formally established due to parental sample unavailability. Trio extension is the highest-leverage near-term intervention to refine these classifications. In families where no indications of potential parental germline mosaicism were present (e.g., siblings with identical variants or isolated variant reads in the parents), the inheritance pattern was assigned based on trio WES data, without additional tissue testing. The rate of de novo variants detected within our cohort (37.3%) is consistent with previous WES studies, where a slightly higher proportion of de novo variants was estimated for cohorts consisting exclusively of neurodevelopmental disorders [62]. Even though some studies report up to 20% of apparently de novo variants arising from parental mosaicism [63], our rates are consistent with the high enrichment of our cohort in severe syndromic neurodevelopmental phenotypes. Multi-sibling family studies may help identify parental mutational load and more accurately predict recurrence risks.

Another limitation is relevant to the methodology and data analysis used within diagnostic settings: a cautious approach was adopted for CNV reporting from ES data. Expanding our analytical framework to include more sensitive CNV detection from exome data could further improve the diagnostic yield of this cohort. Improved read-depth and exon-level CNV calling pipelines can identify clinically relevant deletions and duplications that may otherwise remain unrecognized. Integrating these CNV calls with detailed phenotypic information would be especially valuable and may help resolve currently unsolved cases. The exclusive use of short-read ES and clinical panels means that several classes of pathogenic variation, including structural variants outside captured exons, non-coding regulatory variants, and repeat expansions, were not systematically interrogated. Complementary modalities (short-read genome sequencing, long-read sequencing, RNA-seq, methylome analysis) are expected to uncover additional findings, particularly in the neurodevelopmental-only subgroup where the present yield is lowest.

Finally, a limitation shared with diagnostic sequencing in general is the high burden of VUS, particularly within highly heterogeneous gene groups such as those encoding structural collagens. As demonstrated by recent large-scale genomic analyses, successful reclassification of VUS typically relies on three critical avenues: (1) robust segregation studies to establish phenotype–genotype correlation within families; (2) the development and integration of high-throughput in vitro functional assays or RNA sequencing to actively test variant impact (e.g., assessing splicing defects or protein stability); and (3) systematic, periodic genome reanalysis utilizing updated computational tools and growing population allele databases. Future efforts must prioritize the integration of RNA diagnostics to actively resolve these ambiguous variants and maximize the clinical utility of the genomic medicine service.

## 5. Conclusions

We present the first systematic, mutational-level characterization of a Cypriot Mendelian disease cohort of substantial size, establishing a critical baseline diagnostic-yield reference for the local genomic diagnostic services. In a population historically characterized by geographic isolation, endogamy, and distinct founder effects, defining this baseline is a necessary first step toward implementing equitable precision medicine.

The high rate of novel variants in this study highlights the historical under-representation of Cypriot and Eastern Mediterranean populations in global genomic databases such as gnomAD and ClinVar. Because this lack of reference data actively hinders variant interpretation, we strongly advocate for the systematic submission of population-specific variants, particularly those supported by functional or segregation data, to public repositories. Enriching these databases will reduce diagnostic ambiguity and accelerate interpretation for future Mediterranean cohorts.

Furthermore, the clustering of diagnoses within specific functional gene families suggests that targeted, phenotype-driven reanalysis pipelines could streamline the diagnostic workflow. As sequencing costs decline, prioritizing these high-yield gene networks can make clinical genomic analysis more efficient. Ultimately, establishing a robust local genetic baseline will improve current diagnostics and provide the foundation for population-specific screening and precision medicine initiatives in Cyprus.

## Figures and Tables

**Table 1:** Diagnostic yield as a total and per category.

Group Category	Without Findings	with Finding	No. of Referrals	%Diagnostic Rate
NDD with congenital anomalies/dysmorphism	290	57	347	16.4
isolated NDD	71	5	76	6.6
isolated (single-system) congenital anomaly	106	29	135	21.5
multisystem	96	28	124	22.6
epilepsy/NDD	5	5	10	50.0
Total	568	124	692	17.9

**Table 2:** Novel variants identified in the cohort (*n* = 52).

Gene	Coding HGVS	Variant Type	Zygosity	Inheritance	CL	OMIM Disease#
*ADGRG1*	NC_000016.9:g.57697364_57697715del; NM_201525.4:c.1675C>T (known variant)	Exonic deletion, missense	CH	Biparental	LP, P	606854
*ADAMTSL4*	NC_000001.10:g.150530949_150531641del	Exonic deletion	HO	Biparental	P	225200; 225100
*ADGRL1*	NM_014921.5:c.1328_1331del	Frameshift	H	Maternally Inherited	LP	620065
*ANKRD11*	NM_013275.6:c.4406G>A	Nonsense	H	De novo	LP	148050
*ASH1L*	NM_018489.3:c.2136_2140del	Frameshift	H	Parental mosaicism	LP	617796
*ATP1A3*	NM_001256214.2:c.2155G>C	Missense	H	De novo	P	619606
*BICRA*	NM_015711.3:c.2917C>T	Nonsense	H	De novo	LP	619325
*BRAF*	NM_004333.6:c.1912G>A	Missense	H	Maternally inherited	LP	613706
*CDC42BPB*	NM_006035.4:c.2726+1G>A	Splicing	H	De novo	LP	619841
*COL10A1*	NM_000493.4:c.1781C>T	Missense	H	Maternally inherited	LP	156500
*COL4A1*	NM_001845.6:c.96dup	Frameshift	H	Maternally inherited	LP	175780
*COL5A1*	NM_000093.5:c.4136del	Nonsense	H	De novo	P	130000
*COL5A1*	NM_001278074.1:c.4457G>A	Missense	H	De novo	P	130000; 619329
*COL11A1*	NM_001854.4:c.604G>A	Missense	H	N/A	VUS	618533
*CREBBP*	NM_004380.3:c.5368T>C	Missense	H	De novo	LP	618332
*CUL3*	NM_003590.5:c.1322del	Frameshift	H	Paternally inherited	LP	619239
*CUL4B*	NM_001079872.2:c.1255del	Frameshift	HE	Maternally inherited	LP	300354
*DDX3X*	NM_001356.5:c.1000G>T	Nonsense	H	De novo	LP	300958
*DDX3X*	NM_001356.5:c.1085G>A	Missense	HE	Maternally inherited	LP	300958
*DLL3*	NM_203486.3:c.1097_1098delinsAT; NM_203486.3:c.133dup	Missense, Indel	CH	Biparental	VUS, P	277300
*DSG1*	NM_001942.4:c.1165del	Frameshift	H	Paternally inherited	P	148700
*FBXO11*	NM_001190274.2:c.2694_2695insGA	Frameshift	H	N/A	LP	618089; 619694
*GRIN2B*	NM_000834.5:c.1936T>A	Missense	H	De novo	VUS	613970; 616139
*KCNJ3*	NM_002239.4:c.634A>G	Missense	H	De novo	VUS	No OMIM disease
*KDM5C*	NM_004187.5:c.3267_3270del	Frameshift	H	De novo	LP	300534
*LMBRD2*	NM_001007527.2:c.1394C>G	Nonsense	H	N/A	VUS	618089; 619694
*MYO15A*	NM_016239.4:c.10480C>T (novel); NM_016239.4:c.8457C>G (known variant)	Nonsense, Nonsense	CH	Biparental	LP, P	600316
*SLC17A5*	NM_012434.5:c.701G>A	Missense	HO	Biparental	VUS	600060; 269920
*NAA15*	NM_057175.5:c.908_915dup	Frameshift	H	De novo	P	617787
*OTUD5*	NM_001136157.2:c.773T>A	Missense	HE	Maternally inherited	VUS	301056
*PAX6*	NM_001258462.3:c.399+1G>C	Splicing	H	De novo	P	106210
*PAX6*	NM_001368894.2:c.416G>A	Missense	H	Paternally inherited	LP	106210
*PGM2L1*	NC_000001.10:g.73978096_74062632del	Exonic deletion	HO	Biparental	P	620191
*PKHD1*	NM_138694.4:c.10072G>T (novel); NM_138694.4:c.5060T>C (known)	Missense, Missense	CH	Biparental	VUS, P	263200
*PLS1*	NM_001145319.2:c.1774C>G	Missense	H	Maternally inherited	VUS	618787
*POGZ*	NC_000001.10:g.151384091_151384883del	Exonic deletion	H	De novo	LP	616364
*PPP3CA*	NM_000944.5:c.1387_1397dup	Frameshift	H	De novo	LP	617711; 618265
*SCN2A*	NM_001040143.2:c.832T>C	Missense	H	De novo	P	613721
*SETD2*	NM_014159.7:c.3964C>T	Nonsense	H	De novo	P	616831
*SIN3A*	NM_001145358.2:c.723del	Frameshift	H	De novo	P	613406
*SMAD3*	NM_005902.4:c.1135G>C	Missense	H	Paternally inherited	LP	613795
*SMAD6*	NM_005585.5:c.704T>C	Missense	H	N/A	VUS	614823
*STRC*	Deletion (exons 19 and 20)	Exonic deletion	HO	Biparental	P	603710
*SYNGAP1*	NM_006772.3:c.926dup	Frameshift	H	De novo	LP	612621
*TLK2*	NM_006852.6:c.1000_1002delinsTTTATAGAAATTTATAGAAATTCCGTTGTCT	Frameshift	H	De novo	LP	618050
*TONSL*	NM_013432.5:c.1024G>T; NM_013432.5:c.418A>T	Nonsense, Missense	CH	Biparental	LP, VUS	271510
*UBA2*	NM_005499.3:c.70G>A	Missense	H	Paternally inherited	VUS	619959
*UBE3A*	NM_130839.5:c.1665_1674del	Frameshift	H	Maternally inherited	LP	105830
*WNT1*	NM_005430.4:c.436G>A	Missense	H	N/A	LP	615221
*ZNF469*	NM_001367624.2:c.1069del; (novel) NM_001367624.2:c.11577_11580dup (known)	Frameshift, Frameshift	CH	Biparentally inherited	LP, LP	605130; 229200

P, Pathogenic; LP, Likely pathogenic; VUS, Variant of uncertain significance; HGVS, Human Genome Variation Society nomenclature; OMIM, Online Mendelian Inheritance in Man; CH, Compound Heterozygous; H, Heterozygous; HO, Homozygous; HE, Hemizygous; CL: classification.

## Data Availability

The original contributions presented in this study are included in the article/Supplementary Material. Further inquiries can be directed to the corresponding author. The reported variants were submitted to the freely accessible, public database of human genetic variants ClinVar (www.ncbi.nlm.nih.gov/clinvar/ (ClinVar accession numbers: SCV007615186-SCV007615318).

## Supplementary Materials

The following supporting information can be downloaded at: https://www.mdpi.com/article/10.3390/genes17080863/s1, Table S1: Known/previously reported variants.

## Abbreviations

The following abbreviations are used in this manuscript:

ACGS	Association for Clinical Genomic Science
AD	Autosomal dominant
ADHD	Attention Deficit Hyperactivity Disorder
AR	Autosomal recessive
ASD	Autism Spectrum Disorders
CA	Congenital Disorder
CES	Clinical Exome Sequencing
CING	Cyprus Institute of Neurology and Genetics
CNV	Copy number variant
DD	Developmental Delay
ES	Exome sequencing
GATK	Genome Analysis Toolkit
HGVS	Human Genome Variation Society nomenclature
ID	Intellectual Disability
LP	Likely pathogenic
NDDs	Neurodevelopmental disorders
OMIM	Online Mendelian Inheritance in Man
P	Pathogenic
UTR	Untranslated region
VUS	Variant of uncertain significance
WES	Whole-Exome sequencing
XLD	X-linked dominant
XLR	X-linked recessive

## References

[1] Boyle B., Addor M.C., Arriola L., Barisic I., Bianchi F., Csaky-Szunyogh M., de Walle H.E.K., Dias C.M., Draper E., Gatt M. (2018). Estimating Global Burden of Disease due to congenital anomaly: An analysis of European data. Arch. Dis. Child. Fetal Neonatal Ed..

[2] Rosano A., Botto L.D., Botting B., Mastroiacovo P. (2000). Infant mortality and congenital anomalies from 1950 to 1994: An international perspective. J. Epidemiol. Community Health.

[3] World Health Organization (2023). Congenital Disorders. https://www.who.int/news-room/fact-sheets/detail/birth-defects.

[4] Quinn M. (2024). Congenital anomalies and their impact on child development. J. Clin. Image Case Rep..

[5] Chaves T.F., Ocampos M., Barbato I.T., de Camargo Pinto L.L., de Luca G.R., Barbato Filho J.H., Bernardi P., Costa Netto Muniz Y., Francesca Maris A. (2024). A cohort study of neurodevelopmental disorders and/or congenital anomalies using high resolution chromosomal microarrays in southern Brazil highlighting the significance of ASD. Sci. Rep..

[6] AlMutiri R., Malta M., Shevell M.I., Srour M. (2023). Evaluation of Individuals with Non-Syndromic Global Developmental Delay and Intellectual Disability. Children.

[7] Deciphering Developmental Disorders S. (2017). Prevalence and architecture of de novo mutations in developmental disorders. Nature.

[8] Fu J.M., Satterstrom F.K., Peng M., Brand H., Collins R.L., Dong S., Wamsley B., Klei L., Wang L., Hao S.P. (2022). Rare coding variation provides insight into the genetic architecture and phenotypic context of autism. Nat. Genet..

[9] Wang T., Kim C.N., Bakken T.E., Gillentine M.A., Henning B., Mao Y., Gilissen C., Consortium S., Nowakowski T.J., Eichler E.E. (2022). Integrated gene analyses of de novo variants from 46,612 trios with autism and developmental disorders. Proc. Natl. Acad. Sci. USA.

[10] Satterstrom F.K., Kosmicki J.A., Wang J., Breen M.S., De Rubeis S., An J.Y., Peng M., Collins R., Grove J., Klei L. (2020). Large-Scale Exome Sequencing Study Implicates Both Developmental and Functional Changes in the Neurobiology of Autism. Cell.

[11] De Rubeis S., He X., Goldberg A.P., Poultney C.S., Samocha K., Cicek A.E., Kou Y., Liu L., Fromer M., Walker S. (2014). Synaptic, transcriptional and chromatin genes disrupted in autism. Nature.

[12] Chundru V.K., Zhang Z., Walter K., Lindsay S.J., Danecek P., Eberhardt R.Y., Gardner E.J., Malawsky D.S., Wigdor E.M., Torene R. (2024). Federated analysis of autosomal recessive coding variants in 29,745 developmental disorder patients from diverse populations. Nat. Genet..

[13] Ramaswamy S., Jain R., El Naofal M., Halabi N., Yaslam S., Taylor A., Tayoun A.A. (2022). Middle Eastern Genetic Variation Improves Clinical Annotation of the Human Genome. J. Pers. Med..

[14] Bhattacharya R., Chen N., Shim I., Kuwahara H., Gao X., Alkuraya F.S., Fahed A.C. (2023). Massive underrepresentation of Arabs in genomic studies of common disease. Genome Med..

[15] Lipinski R.J., Krauss R.S. (2023). Gene-environment interactions in birth defect etiology: Challenges and opportunities. Curr. Top. Dev. Biol..

[16] Jin S.C., Homsy J., Zaidi S., Lu Q., Morton S., DePalma S.R., Zeng X., Qi H., Chang W., Sierant M.C. (2017). Contribution of rare inherited and de novo variants in 2871 congenital heart disease probands. Nat. Genet..

[17] Mone F., Eberhardt R.Y., Morris R.K., Hurles M.E., McMullan D.J., Maher E.R., Lord J., Chitty L.S., Giordano J.L., Wapner R.J. (2021). COngenital heart disease and the Diagnostic yield with Exome sequencing (CODE) study: Prospective cohort study and systematic review. Ultrasound Obstet. Gynecol..

[18] Reilly K., Sonner S., McCay N., Rolnik D.L., Casey F., Seale A.N., Watson C.J., Kan A., Lai T.H.T., Chung B.H.Y. (2024). The incremental yield of prenatal exome sequencing over chromosome microarray for congenital heart abnormalities: A systematic review and meta-analysis. Prenat. Diagn..

[19] Srivastava S., Love-Nichols J.A., Dies K.A., Ledbetter D.H., Martin C.L., Chung W.K., Firth H.V., Frazier T., Hansen R.L., Prock L. (2019). Meta-analysis and multidisciplinary consensus statement: Exome sequencing is a first-tier clinical diagnostic test for individuals with neurodevelopmental disorders. Genet. Med..

[20] Manickam K., McClain M.R., Demmer L.A., Biswas S., Kearney H.M., Malinowski J., Massingham L.J., Miller D., Yu T.W., Hisama F.M. (2021). Exome and genome sequencing for pediatric patients with congenital anomalies or intellectual disability: An evidence-based clinical guideline of the American College of Medical Genetics and Genomics (ACMG). Genet. Med..

[21] Neuens S., Soblet J., Penninckx A., Detry C., Badoer C., Desmyter L., Peyrassol X., Wilkin F., Busson A., Bruneau M. (2025). Diagnostic yield of clinical exome sequencing in 868 children with neurodevelopmental disorders. Eur. J. Med. Genet..

[22] Emms A., Castleman J., Allen S., Williams D., Kinning E., Kilby M. (2022). Next Generation Sequencing after Invasive Prenatal Testing in Fetuses with Congenital Malformations: Prenatal or Neonatal Investigation. Genes.

[23] van der Ven A.T., Connaughton D.M., Ityel H., Mann N., Nakayama M., Chen J., Vivante A., Hwang D.Y., Schulz J., Braun D.A. (2018). Whole-Exome Sequencing Identifies Causative Mutations in Families with Congenital Anomalies of the Kidney and Urinary Tract. J. Am. Soc. Nephrol..

[24] Taniguchi K., Hasegawa F., Okazaki Y., Hori A., Ogata-Kawata H., Aoto S., Migita O., Kawai T., Nakabayashi K., Okamura K. (2025). Approaches to Evaluate Whole Exome Sequencing Data That Incorporate Genetic Intolerance Scores for Congenital Anomalies, Including Intronic Regions Adjacent to Exons. Mol. Genet. Genom. Med..

[25] Heraclides A., Bashiardes E., Fernandez-Dominguez E., Bertoncini S., Chimonas M., Christofi V., King J., Budowle B., Manoli P., Cariolou M.A. (2017). Y-chromosomal analysis of Greek Cypriots reveals a primarily common pre-Ottoman paternal ancestry with Turkish Cypriots. PLoS ONE.

[26] Moutsouri I., Keravnou A., Manoli P., Bertoncini S., Michailidou K., Christofi V., Xenophontos S., Cariolou M.A., Bashiardes E. (2021). Comparative Y-chromosome analysis among Cypriots in the context of historical events and migrations. PLoS ONE.

[27] Georgiou T., Petrou P.P., Malekkou A., Ioannou I., Gavatha M., Skordis N., Nicolaidou P., Savvidou I., Athanasiou E., Ourani S. (2024). Inherited metabolic disorders in Cyprus. Mol. Genet. Metab. Rep..

[28] Antoniades A., Chi J., Brown C., Vogazianos P., Charalambous E., Vrachnos D., Aristodimou A., Miltiadous A., Papaloizou A., Koumouli A. (2026). Population genetic variation characterised through serial independent pool-seq: The Cyprus Genome Project. Sci. Rep..

[29] Ewels P., Magnusson M., Lundin S., Kaller M. (2016). MultiQC: Summarize analysis results for multiple tools and samples in a single report. Bioinformatics.

[30] Pedersen B.S., Quinlan A.R. (2017). Who’s Who? Detecting and Resolving Sample Anomalies in Human DNA Sequencing Studies with Peddy. Am. J. Hum. Genet..

[31] Manichaikul A., Mychaleckyj J.C., Rich S.S., Daly K., Sale M., Chen W.M. (2010). Robust relationship inference in genome-wide association studies. Bioinformatics.

[32] Plagnol V., Curtis J., Epstein M., Mok K.Y., Stebbings E., Grigoriadou S., Wood N.W., Hambleton S., Burns S.O., Thrasher A.J. (2012). A robust model for read count data in exome sequencing experiments and implications for copy number variant calling. Bioinformatics.

[33] Durkie M., Cassidy E.J., Berry I., Owens M., Turnbull C., Scott R.H., Taylor R.W., Deans Z.C., Ellard S., McMullan D.J. (2024). ACGS Best Practice Guidelines for Variant Classification in Rare Disease 2024.

[34] Richards S., Aziz N., Bale S., Bick D., Das S., Gastier-Foster J., Grody W.W., Hegde M., Lyon E., Spector E. (2015). Standards and guidelines for the interpretation of sequence variants: A joint consensus recommendation of the American College of Medical Genetics and Genomics and the Association for Molecular Pathology. Genet. Med..

[35] Riggs E.R., Andersen E.F., Cherry A.M., Kantarci S., Kearney H., Patel A., Raca G., Ritter D.I., South S.T., Thorland E.C. (2020). Technical standards for the interpretation and reporting of constitutional copy-number variants: A joint consensus recommendation of the American College of Medical Genetics and Genomics (ACMG) and the Clinical Genome Resource (ClinGen). Genet. Med..

[36] Untergasser A., Cutcutache I., Koressaar T., Ye J., Faircloth B.C., Remm M., Rozen S.G. (2012). Primer3--new capabilities and interfaces. Nucleic Acids Res..

[37] Lee H., Deignan J.L., Dorrani N., Strom S.P., Kantarci S., Quintero-Rivera F., Das K., Toy T., Harry B., Yourshaw M. (2014). Clinical Exome Sequencing for Genetic Identification of Rare Mendelian Disorders. JAMA.

[38] Andersen P.F., Ek J., Karstensen H.G., Bak M., Gronborg S.W., Hove H.B., Diness B., Hjortshoj T.D., Hammer T.B., Hoi-Hansen C. (2025). Diagnostic yield of whole exome sequencing in a cohort of 825 patients. Eur. J. Med. Genet..

[39] Wright C.F., FitzPatrick D.R., Firth H.V. (2018). Paediatric genomics: Diagnosing rare disease in children. Nat. Rev. Genet..

[40] Merz L.M., Kolvenbach C.M., Wang C., Mertens N.D., Seltzsam S., Mansour B., Zheng B., Schneider S., Schierbaum L., Holzel S. (2025). Trio exome sequencing identifies de novo variants in novel candidate genes in 19.62% of CAKUT families. Genet. Med..

[41] Tal-Ben Ishay R., Shil A., Solomon S., Sadigurschi N., Abu-Kaf H., Meiri G., Flusser H., Michaelovski A., Dinstein I., Golan H. (2021). Diagnostic Yield and Economic Implications of Whole-Exome Sequencing for ASD Diagnosis in Israel. Genes.

[42] Martinez-Granero F., Blanco-Kelly F., Sanchez-Jimeno C., Avila-Fernandez A., Arteche A., Bustamante-Aragones A., Rodilla C., Rodriguez-Pinilla E., Riveiro-Alvarez R., Tahsin-Swafiri S. (2021). Comparison of the diagnostic yield of aCGH and genome-wide sequencing across different neurodevelopmental disorders. npj Genom. Med..

[43] Kritioti E., Theodosiou A., Parpaite T., Alexandrou A., Nicolaou N., Papaevripidou I., Sejourne N., Coste B., Christophidou-Anastasiadou V., Tanteles G.A. (2021). Unravelling the genetic causes of multiple malformation syndromes: A whole exome sequencing study of the Cypriot population. PLoS ONE.

[44] Del Gobbo G.F., Boycott K.M. (2025). The additional diagnostic yield of long-read sequencing in undiagnosed rare diseases. Genome Res..

[45] Koyutourk B., Ergoren M.C. (2026). Rare diseases in the Turkish-Cypriot community: A nationwide study. J. Community Genet..

[46] Fanidis D., Moutsouri E., Malatras A., Shuldiner A.R., Alkelai A., Polydorou C., Papagregoriou G., Deltas C. (2026). Medically actionable DNA variants have a prevalence of 5% in the Cyprus genetic landscape. Genomics.

[47] Chioza B., Osei-Lah A., Wilkie H., Nashef L., McCormick D., Asherson P., Makoff A.J. (2002). Suggestive evidence for association of two potassium channel genes with different idiopathic generalised epilepsy syndromes. Epilepsy Res..

[48] Feng Y.-C.A., Howrigan D.P., Abbott L.E., Tashman K., Cerrato F., Singh T., Heyne H., Byrnes A., Churchhouse C., Watts N. (2019). Ultra-Rare Genetic Variation in the Epilepsies: A Whole-Exome Sequencing Study of 17,606 Individuals. Am. J. Hum. Genet..

[49] Li J., Mei S., Mao X., Wan L., Wang H., Xiao B., Song Y., Gu W., Liu Y., Long L. (2024). De novo variants in KCNJ3 are associated with early-onset epilepsy. J. Med. Genet..

[50] Lee H., Lin M.C., Kornblum H.I., Papazian D.M., Nelson S.F. (2014). Exome sequencing identifies de novo gain of function missense mutation in KCND2 in identical twins with autism and seizures that slows potassium channel inactivation. Hum. Mol. Genet..

[51] Lin M.A., Cannon S.C., Papazian D.M. (2018). Kv4.2 autism and epilepsy mutation enhances inactivation of closed channels but impairs access to inactivated state after opening. Proc. Natl. Acad. Sci. USA.

[52] Zhang Y., Tachtsidis G., Schob C., Koko M., Hedrich U.B.S., Lerche H., Lemke J.R., van Haeringen A., Ruivenkamp C., Prescott T. (2021). KCND2 variants associated with global developmental delay differentially impair Kv4.2 channel gating. Hum. Mol. Genet..

[53] Wardeh A., Jackson T., Nelson B., Ernst C., Theroux J.F., Al-Hertani W., Sobering A.K., Maj M.C. (2018). Identification of a de novo case of COL5A1-related Ehlers-Danlos syndrome in an infant in the West Indies leading to improved targeted clinical care. Clin. Case Rep..

[54] Scocchia A., Kangas-Kontio T., Irving M., Hero M., Saarinen I., Pelttari L., Gall K., Valo S., Huusko J.M., Tallila J. (2021). Diagnostic utility of next-generation sequencing-based panel testing in 543 patients with suspected skeletal dysplasia. Orphanet J. Rare Dis..

[55] Hadjipanteli A., Theodosiou A., Papaevripidou I., Evangelidou P., Alexandrou A., Salameh N., Kallikas I., Kakoullis K., Frakala S., Oxinou C. (2024). Sodium Channel Gene Variants in Fetuses with Abnormal Sonographic Findings: Expanding the Prenatal Phenotypic Spectrum of Sodium Channelopathies. Genes.

[56] Ma R., Duan Y., Zhang L., Qi X., Zhang L., Pan S., Gao L., Wang C., Wang Y. (2022). SCN1A-Related Epilepsy: Novel Mutations and Rare Phenotypes. Front. Mol. Neurosci..

[57] Kurekci F., Akif Kilic M., Akbas S., Avci R., Oney C., Dilruba Aslanger A., Maras Genc H., Aydinli N., Pembegul Yildiz E. (2024). Voltage-gated sodium channel epilepsies in a tertiary care center: Phenotypic spectrum with correlation to predicted functional effects. Epilepsy Behav..

[58] Bjornsson H.T. (2015). The Mendelian disorders of the epigenetic machinery. Genome Res..

[59] Skalicka P., Porter L.F., Brejchova K., Malinka F., Dudakova L., Liskova P. (2020). Brittle cornea syndrome: Disease-causing mutations in ZNF469 and two novel variants identified in a patient followed for 26 years. Biomed. Pap. Med. Fac. Univ. Palacky Olomouc Czech Repub..

[60] Amir R.E., Van den Veyver I.B., Wan M., Tran C.Q., Francke U., Zoghbi H.Y. (1999). Rett syndrome is caused by mutations in X-linked MECP2, encoding methyl-CpG-binding protein 2. Nat. Genet..

[61] Di Muro E., Petracca A., Castori M., Palumbo O. (2025). Gonadal Mosaicism for an *ASH1L* Intragenic Deletion Makes a Bridge Between MRD52 and 1q22 Microdeletion. Am. J. Med. Genet..

[62] Brunet T., Jech R., Brugger M., Kovacs R., Alhaddad B., Leszinski G., Riedhammer K.M., Westphal D.S., Mahle I., Mayerhanser K. (2021). De novo variants in neurodevelopmental disorders-experiences from a tertiary care center. Clin. Genet..

[63] Lee M., Lui A.C.Y., Chan J.C.K., Doong P.H.L., Kwong A.K.Y., Mak C.C.Y., Li R.H.W., Kan A.S.Y., Chung B.H.Y. (2023). Revealing parental mosaicism: The hidden answer to the recurrence of apparent de novo variants. Hum. Genom..

